# OVCAR-3 Spheroid-Derived Cells Display Distinct Metabolic Profiles

**DOI:** 10.1371/journal.pone.0118262

**Published:** 2015-02-17

**Authors:** Kathleen A. Vermeersch, Lijuan Wang, Roman Mezencev, John F. McDonald, Mark P. Styczynski

**Affiliations:** 1 School of Chemical & Biomolecular Engineering, Georgia Institute of Technology, Atlanta, Georgia, United States of America; 2 Ovarian Cancer Institute and School of Biology, Georgia Institute of Technology, Atlanta, Georgia, United States of America; University of Nebraska Medical Center, UNITED STATES

## Abstract

**Introduction:**

Recently, multicellular spheroids were isolated from a well-established epithelial ovarian cancer cell line, OVCAR-3, and were propagated *in vitro*. These spheroid-derived cells displayed numerous hallmarks of cancer stem cells, which are chemo- and radioresistant cells thought to be a significant cause of cancer recurrence and resultant mortality. Gene set enrichment analysis of expression data from the OVCAR-3 cells and the spheroid-derived putative cancer stem cells identified several metabolic pathways enriched in differentially expressed genes. Before this, there had been little previous knowledge or investigation of systems-scale metabolic differences between cancer cells and cancer stem cells, and no knowledge of such differences in ovarian cancer stem cells.

**Methods:**

To determine if there were substantial metabolic changes corresponding with these transcriptional differences, we used two-dimensional gas chromatography coupled to mass spectrometry to measure the metabolite profiles of the two cell lines.

**Results:**

These two cell lines exhibited significant metabolic differences in both intracellular and extracellular metabolite measurements. Principal components analysis, an unsupervised dimensional reduction technique, showed complete separation between the two cell types based on their metabolite profiles. Pathway analysis of intracellular metabolomics data revealed close overlap with metabolic pathways identified from gene expression data, with four out of six pathways found enriched in gene-level analysis also enriched in metabolite-level analysis. Some of those pathways contained multiple metabolites that were individually statistically significantly different between the two cell lines, with one of the most broadly and consistently different pathways, arginine and proline metabolism, suggesting an interesting hypothesis about cancerous and stem-like metabolic phenotypes in this pair of cell lines.

**Conclusions:**

Overall, we demonstrate for the first time that metabolism in an ovarian cancer stem cell line is distinct from that of more differentiated isogenic cancer cells, supporting the potential importance of metabolism in the differences between cancer cells and cancer stem cells.

## Introduction

Ovarian cancer is the deadliest gynecological cancer and the 5^th^ leading cause of cancer-related death in United States women. An estimated 15,500 U.S. women died from ovarian cancer in 2012 [1]. Even though response to the first-line treatment is high, most patients (50–75%) relapse after the treatment [2]. Recently, side populations of cancer cells with ABC-transporter activity and ability to efflux certain compounds have been identified in many different types of tumors, including ovarian cancer [3–5]. These cells are referred to as cancer stem cells because of their many unique properties: they have self-renewal and differentiation capabilities and exhibit resistance to the effects of radiation and anticancer drugs [6]. Based on these properties, they are suspected as a primary cause of cancer recurrence [7, 8].

We have previously isolated an anoikis-independent subpopulation of a widely-used ovarian cancer cell (OCC) line, OVCAR-3, to establish an ovarian cancer stem cell (OCSC) line [8]. These spheroid-derived cells were shown to demonstrate numerous characteristics of cancer stem cells, including self-renewal, the ability to produce differentiated progeny, increased expression of genes associated with cancer stem cells, higher invasiveness, migration potential, and enhanced chemoresistance. Transcriptional analysis identified changes in various signalling pathways including TGF-beta-dependent induction of epithelial-to-mesenchymal transition, regulation of lipid metabolism, and NOTCH and Hedgehog signaling. In addition, eleven pathways associated with metabolism were identified as being enriched with genes differentially expressed between OCSCs and OCCs, suggesting a potential role of metabolic differences as a cause or consequence of the phenotypic differences between the two cell types.

Since transcriptional changes do not always directly translate to changes in metabolism, we sought to directly test the prediction that OCCs and isogenic OCSCs may display significant differences in metabolism. We tested this prediction using metabolomics, the systems-scale analysis of the levels of small-molecule biochemical (metabolic) intermediates in the cell. Metabolomics is a field that is increasingly being brought to bear on the study of cancer, with promising results including insights into disease mechanisms as well as the identification of circulating biomarkers for potential use in diagnostics [9–12]. Metabolomics has the potential to reveal key intracellular pathways or metabolites and their related enzymes in OCSCs that may serve as potential therapeutic targets, as well as secreted or intracellular metabolites that may find potential use in diagnostic tests. In fact, metabolism has previously been suggested as a potential therapeutic target for cancer stem cells [13–15]. However, to date metabolomics has rarely been exploited to study cancer stem cells. To our knowledge, the only reported applications of metabolomics in this context was its use to study the differences between tumors derived from fresh glioma stem cells and cultured glioma stem cells *in vivo* using nuclear magnetic resonance spectroscopy [16], a recently published report on breast cancer stem cells’ reliance on glycolysis [17], and recent isolation of a colon cancer stem cell line with metabolomic characterization [18]. There is also increasing recent interest in cancer stem cell metabolism through approaches other than metabolomics, with many of the discoveries focused on enhanced glycolysis [19–30].

In this study, we analyzed the intracellular and extracellular metabolomic profiles of both ovarian cancer cell types *in vitro* over two days using two dimensional gas chromatography-mass spectrometry (GCxGC-MS), the first-ever metabolomic comparison between ovarian cancer cells and their isogenic cancer stem cells. Consistent with gene expression-driven predictions, we identified significant differences between the metabolism of these two cell types. Our findings are consistent with the hypothesis that metabolic changes may contribute to the functional differences between ovarian cancer stem cells and their more differentiated progeny that represent the majority of bulk tumor tissue. More generally, our findings are consistent with the mounting body of evidence that altered metabolism is one of the hallmarks of cancer [31].

## Methods

### Cell culture

The OVCAR-3 cell line [32] was obtained from the Developmental Therapeutics Program (DTP) of the National Cancer Institute (NCI). The OVCAR-3 ovarian cancer cells (OCCs) were cultured in R10 medium: RPMI-1640 (Cellgro, Mediatech Inc., Manassas, VA) supplemented with 10% fetal bovine serum (FBS, Invitrogen, Grand Island, NY) and 1% antibiotic-antimycotic solution (Cellgro, Mediatech Inc., Manassas, VA). Authenticity of the OVCAR-3 cell line was confirmed using short tandem repeat profiling performed by IDEXX RADIL (Columbia, MO) in October 2013. Cells were grown until confluence and subcultured at a ratio of 1:4. Immediately before any time-course sampling began, OCCs were passaged and seeded in 6-well plates (Greiner Bio-One, Monroe, NC) at a density of 3x10^5^ cells/well and incubated for 24 hours in R10 medium to allow the cells to attach and recover; the medium was removed, wells were washed once with PBS, and then fresh R10 medium was added to begin the experiment.

Ovarian cancer stem cells (OCSCs) we previously derived [8] from the OVCAR-3 cell line were cultured as previously described [8]. Briefly, the OCSCs were cultured in ultra-low attachment petri dishes (Corning Incorporated, Corning, NY) in stem cell medium: DMEM/F12 (1:1) (Cellgro, Mediatech Inc., Manassas, VA) supplemented with 0.4% bovine serum albumin (BSA, Sigma-Aldrich, St. Louis, MO), 20 ng/mL epidermal growth factor (EGF, Invitrogen, Grand Island, NY), 10 ng/mL basic fibroblast growth factor (bFGF, Sigma-Aldrich, St. Louis, MO), 5 μg/mL insulin (Sigma-Aldrich, St. Louis, MO), and 1% antibiotic-antimycotic solution (Cellgro, Mediatech Inc., Manassas, VA). The spheroids were dissociated and re-seeded at a density of 10^5^ cells/mL each week. To begin the time-course experiment, OCSCs were dissociated and seeded into ultra-low attachment 6-well plates (Corning Incorporated, Corning, NY) containing 2 mL of fresh stem cell medium at a density of 3x10^5^ cells/well. Both the OCC and OCSC experiments were performed in biological triplicates.

### Sampling Protocols

Samples were taken at 0 minutes, 15 minutes, 30 minutes, 8 hours, 24 hours, and 48 hours to capture both short- and long-term metabolic profiles. For OCCs, medium was removed and snap frozen in liquid nitrogen for extracellular analysis. Cells were then quickly washed with 1mL PBS at 37°C, which was aspirated off, and then 700 μL of 80:20 methanol/water solution at -80°C was added immediately. The plate was then incubated at -80°C for 15 minutes. After incubation, remaining cellular debris were harvested using a cell scraper (BD Falcon, San Jose, CA) for intracellular analysis. For OCSC cells, the media-cell mixture was transferred to a 15 mL centrifuge tube and centrifuged at 1,900 *g* for 30 seconds at room temperature. The supernatant was removed and snap frozen in liquid nitrogen for extracellular analysis. The cell pellet was quickly resuspended in 1mL PBS at 37°C and then immediately centrifuged again at 1,900 *g* for 30 seconds at room temperature. The wash solution was discarded and the cell pellet was resuspended in 700 μL of 80:20 methanol/water at -80°C. The samples were then incubated at -80°C for 15 minutes.

For both cell types, the intracellular solution was then transferred to a microcentrifuge tube in a cold ethanol bath and centrifuged at 5,000 *g* for 5 minutes at -4°C. The supernatant was retained, and the pellet was subsequently re-extracted twice in 100 μL of the cold 80:20 methanol/water solution, with all supernatants being pooled [33]. Intracellular and extracellular samples were stored at -80°C and -20°C, respectively, until analysis.

### Extracellular Sample Extraction

Immediately before two dimensional gas chromatography-mass spectrometry (GCxGC-MS) analysis, an acetonitrile precipitation was performed on the extracellular samples to remove protein [34]. Briefly, the extracellular samples were thawed on ice and 75 μL was removed for GCxGC-MS analysis. 150 μL of ice-cold acetonitrile was added to the sample, and the sample was vortexed for one minute. The sample was then centrifuged at 21,100 *g* for 7 minutes, and the supernatant removed for GCxGC-MS analysis.

### GCxGC-MS Analysis

Before derivatization, both intracellular and extracellular samples were dried down in a CentriVap at 40°C until completely dry. For the intracellular samples, the volume removed was calculated based on a total of 3x10^5^ cells at the end of derivatization. For the extracellular samples, the entire supernatant from the extracellular extraction was dried down. The samples were derivatized following the protocol laid out by Fiehn, *et*. *al*. [35]. Briefly, 10 μL (extracellular) or 2.5 μL (intracellular) of 40 mg/mL *O*-methylhydroxylamine hydrochloride (MP Biomedicals, LLC, Santa Ana, CA) in pyridine was added to the dried sample and shaken at 1400 rpm for 90 minutes at 30°C. 90 μL (extracellular) or 22.5 μL (intracellular) of *N*-methyl-*N*-(trimethylsilyl) trifluoroacetamide (MSTFA) + 1% trimethylchlorosilane (TMCS) (Thermo Scientific, Lafayette, CO) was then added to the samples which were then shaken at 1400 rpm for 30 minutes at 37°C. Samples were centrifuged at 21,100 *g* for 3 minutes and 50 μL (extracellular) or 15 μL (intracellular) of the supernatant was added to an autosampler vial. Samples were spiked with 0.25 μL (extracellular) or 0.10 μL (intracellular) of a retention time standard solution consisting of fatty acid methyl esters (FAMEs) and an internal standard of nonadecanoic acid methyl ester dissolved in dimethylformamide.

A LECO Pegasus 4D instrument with an Aglient 7683B autosampler, Agilent 7890A gas chromatograph and time-of-flight mass spectrometer (TOF-MS) was used to analyze the samples. The first column was an HP-5, 30 m long x 0.320 mm ID x 0.25 μm film thickness (Agilent, Santa Clara, CA), and the second was an Rtx-200, 2 m long x 0.25 mm ID x 0.25 μm film thickness (Restek, Bellefonte, PA). Specific autosampler, gas chromatography, and mass spectrometry methods can be found in S1 Protocol.

### Data Analysis

Sample runs were first analyzed in ChromaTOF (LECO, St. Joseph, MI) to determine baseline, peak area, and peak identification. Briefly, settings included a baseline offset of 0.5, automatic smoothing, 1^st^ dimension peak width of 10 seconds, 2^nd^ dimension peak width of 0.10 seconds, and a match of 700 required to combine peaks with a minimum signal-to-noise (S/N) of 5 for all subpeaks. Peaks were required to have a S/N of 10 and have a minimum similarity score of 800 before assigning a name. Unique mass was used for area and height calculation.

To align the samples, MetPP (http://metaopen.sourceforge.net/metpp.html) was used [36]. Sample files and a derivatization reagent blank file were uploaded from ChromaTOF. Unknowns were retained during the peak alignment process. The derivatization reagent blank file was used to subtract peaks attributable only to derivatization reagents from the sample files. On-the-fly alignment was used with quality control samples manually selected as the peak list for primary alignment. Peak alignment was performed using the default criteria.

After alignment, further processing of the data was done following the procedure laid out by Dunn, *et*. *al*. [37]. Batch effects were removed from the intracellular data set using LOESS. During LOESS correction, one of the OCC and OCSC 24 hour biological replicates were identified as an outlier and removed. LOESS was also performed on the extracellular data set, but batch effects were amplified in the samples after correction so the original data set was used for subsequent analysis. To remove analytes that were not reproducibly detected, analytes for which more than half of the values were missing in the QC samples or for which the QC samples had a coefficient of variance larger than 0.5 were removed from the data set. Then, missing values were manually corrected using small value correction only if all the values were missing in the biological replicate.

Finally, MetaboAnalyst (http://metaboanalyst.ca/) was used for statistical and enrichment analysis, applying both the statistical analysis and time series analysis modules [38]. Missing values were k-nearest neighbors (KNN) corrected. Data was filtered using the interquantile range method and then log-transformed using generalized logarithm transformation (base 2) and autoscaled.

For enrichment analysis, both metabolite set enrichment analysis (MSEA) and metabolite pathway enrichment analysis (MPEA) yielded similar results, so only MPEA results were considered further. The entire time series was uploaded as discrete data with compound names. Metabolites were properly matched to their HMDB codes before processing the data. Data processing followed the same steps as listed above for missing value imputation and data normalization. The *Homo sapiens* pathway library was used for analysis and an in-house metabolite reference library based on detectable metabolites for our system was uploaded. Global test was used for pathway enrichment analysis, with relative-betweeness centrality as the pathway topology analysis. Pathways with an FDR < 0.05 were considered significantly enriched.

For gene set enrichment analysis (GSEA) of OCSC and OCC gene expression data, .CEL files corresponding to 3 replicated cultures of OCSC and OCC (GeneChip Human Genome U133 Plus 2.0 Array), generated as previously described [8], were processed and normalized by GCRMA method (http://arrayanalysis.org) and used for GSEA (http://www.broadinstitute.org/gsea/index.jsp) without pre-filtering of probe sets using the following parameters: OCC vs. OCSC as categorical phenotypes; signal-to-noise metric; gene set permutation type; curated KEGG gene sets (186 gene sets, Molecular Signatures Database v4.0). Gene sets were considered significantly enriched in a given phenotype if their FDR q value was < 0.20. The dataset employed in this analysis is available in the Gene Expression Omnibus (GEO, http://www.ncbi.nlm.nih.gov/geo/) as series GSE28799.

## Results and Discussion

### OCCs and OCSCs exhibit significant differences on an individual metabolite level

In order to profile cellular metabolism, we performed GCxGC-MS analysis on intracellular and extracellular samples of OCCs and OCSCs. The cell lines were fairly pure cultures based on CD44 expression (OCSCs were at least 85% pure, see S1 Fig.), consistent with our previous characterization of the cell lines [8] and reasonable given the dynamic nature of the epithelial/mesenchymal transition and cell differentiation that play a role in the cancer stem cell phenotype. This analysis of OCCs and OCSCs detected 211 intracellular and 203 extracellular reproducibly measurable analytes overall, including unknowns and analytes that did not map to annotated human metabolites (based on KEGG and HMDB identifications available in MetaboAnalyst). After removing analytes whose changing levels were driven by growth media differences (see S2 Protocol), the resulting intracellular dataset contained 177 unknown and annotated analytes, which included 40 unique identifiable metabolites; the extracellular dataset contained 128 unknown and annotated analytes, which included 46 unique identifiable metabolites. Growth media differences were successfully removed in the intracellular data set, but could not be completely removed from the extracellular data set, as detailed in S2 Protocol. Since the intracellular samples showed no medium-related artifacts and thus allowed for the most direct interpretation, moving forward we focused our attention and analysis solely on those data.

Basic univariate analysis of metabolite levels provided insight into the differences between the two cell types. Of the 40 uniquely identified intracellular metabolites, 27 were significantly different (t-test, all time points, FDR < 0.05) between the two cell types, shown in Table 1, and 19 of these 27 metabolites had a fold change greater than two. Based on these statistical differences, metabolism seems to be considerably altered in OCSCs, though it is impossible to conclude based on metabolite levels alone (without fluxes) whether this may be due to increased or decreased metabolism.

**Table 1:** List of intracellular metabolites statistically significant between OCCs and OCSCs and their fold changes.

**Metabolite**	**p value**	**FDR**	**log_2_(FC)**
Gamma-Aminobutyric acid	2.75E-16	1.10E-14	2.741
D-Psicose	4.96E-10	9.91E-09	1.997
Erythronic acid	4.57E-08	6.10E-07	1.411
Pyrophosphate	6.38E-08	6.38E-07	2.792
Fumaric acid	1.04E-07	8.29E-07	1.751
Erythritol	8.14E-07	5.43E-06	4.243
Putrescine	4.54E-05	2.59E-04	1.739
L-Isoleucine	6.83E-05	3.34E-04	1.187
L-Proline	7.51E-05	3.34E-04	2.736
L-Glutamate	8.80E-05	3.52E-04	1.107
L-Lysine	1.68E-04	6.11E-04	-1.155
Mannitol	2.06E-04	6.86E-04	0.702
Cholesterol	4.01E-04	1.23E-03	0.543
Glycerol	4.62E-04	1.25E-03	-0.754
Pyroglutamic acid	4.69E-04	1.25E-03	0.833
Ethanolamine	6.40E-04	1.51E-03	-0.626
L-Aspartic acid	6.15E-04	1.51E-03	1.265
Uridine 5'-monophosphate	1.09E-03	2.43E-03	0.675
3,7-Dimethyl-3-octanol	2.01E-03	4.00E-03	-0.364
Glycine	1.92E-03	4.00E-03	0.987
L-Malic acid	2.10E-03	4.00E-03	1.611
N-Decane	4.08E-03	7.09E-03	-0.536
Mannose 6-phosphate	4.05E-03	7.09E-03	0.600
Glycerol-3-phosphate	1.43E-02	2.38E-02	0.439
Citric acid	1.62E-02	2.60E-02	-0.267
Hydrogen sulfide	2.00E-02	3.08E-02	0.599
Hexadecane	2.26E-02	3.34E-02	-0.586

Fold changes represent OCC levels relative to OCSC levels of the metabolite.

### Metabolomic analysis reveals distinct metabolic profiles between OCCs and OCSCs

**Fig 1:**
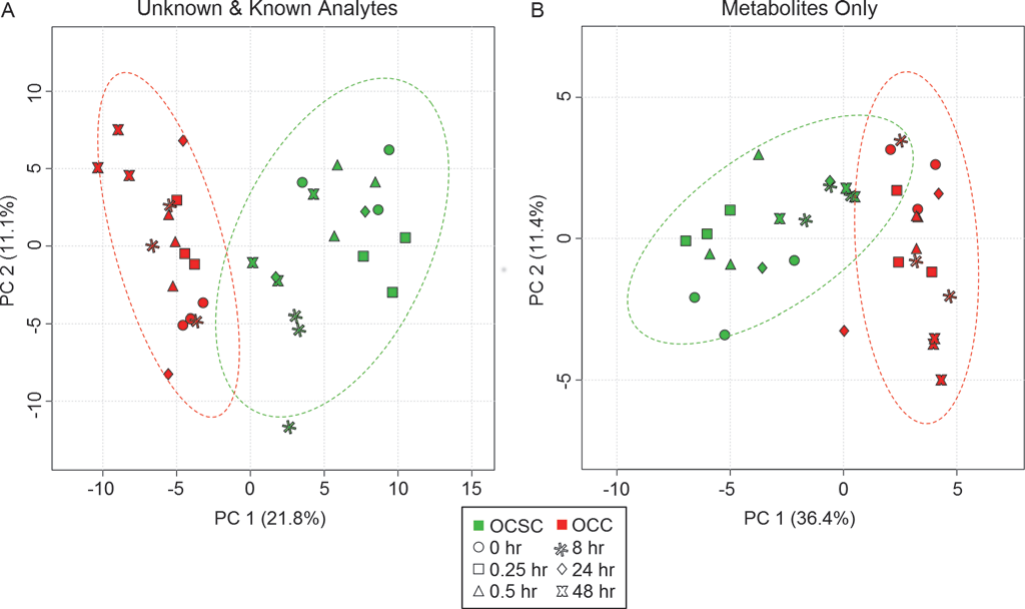
Principal components analysis easily distinguishes between the metabolic profiles of OCCs and OCSCs. Red points are OVCAR-3 cells, and green points are ovarian cancer stem cells at six different time points with three biological replicates at each time point. Principal component (PC) axes include the percentage of variation captured by each axis. A) PC 1 captures the cell type separation for the unknown and annotated analytes, with PC 2 capturing some time point separation. B) Including only the profiles of annotated, identified metabolites shows complete cell type separation in PC 1 and some time point separation in PC 2.

Unsupervised dimensional reduction using principal components analysis (PCA) on the identified metabolite sets showed complete separation for the intracellular metabolic profiles between OCCs and OCSCs, as seen in Fig. 1. PCA of intracellular metabolite measurements showed distinct metabolic profiles between the two cell types captured mostly in principal component (PC) 1 with the second PC providing additional separation, as seen in Fig. 1B. In both of the intracellular plots, the second PC shows some separation between different time points. The fact that this unsupervised method was able to separate sample classes so strongly in the first few PCs suggests the presence of true metabolic differences that are not artifacts of data analysis. PCA based on the entire measured dataset (including unknowns, not just the identified human metabolites) yielded similar separation (Fig. 1A), with a number of unknowns having high loading scores in PCs 1 and 2, suggesting that the differences between the two cell types are not limited to just the well-characterized sections of cellular metabolism.

Analysis of the PCA loadings revealed the metabolites that were the most important in causing the separation seen in the intracellular PCA plots. Fumarate, erythronic acid, psicose, pyrophosphate, gamma-aminobutyric acid, aspartate, isoleucine, uridine 5'-monophosphate, putrescine, and glutamate are most responsible for the cell type separation in the intracellular data set. Interestingly, putrescine, aspartate, glutamate, and fumarate are all involved in the arginine and proline pathway, which was identified in gene set enrichment analysis as significantly enriched in the OCSC phenotype.

**Fig 2:**
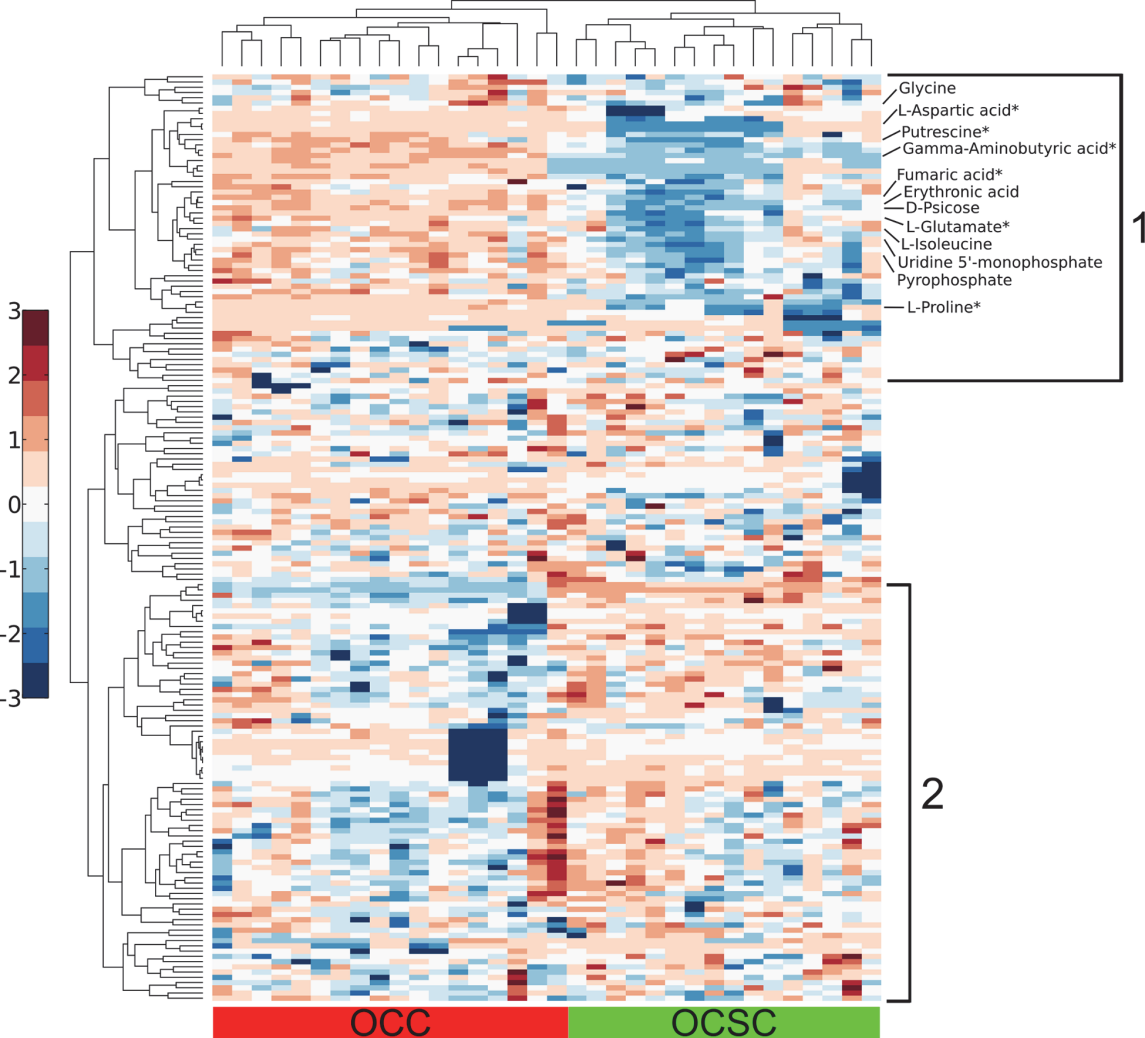
Hierarchical clustering demonstrates clear separation between cell type and major trends in analyte levels. Columns represent hierarchically clustered samples, color-coded according to cell type. Rows represent hierarchically clustered analytes, with the metabolites mentioned in the main text specifically labeled (metabolites from the arginine and proline metabolism pathway are annotated with an *). Metabolite levels are mean-centered and unit-variance on a per-metabolite basis. The intracellular profile consists of two major groups with clearly separate behavior between the cell types. In group 1, OCC analyte levels are generally higher than OCSC analyte levels. Group 2 analyte levels are lower in OCCs than OCSCs.

Hierarchially clustered heatmaps of the intracellular metabolic profiles also revealed distinct patterns, shown in Fig. 2. The samples for OCC and OCSC cluster together tightly, showing the similarity within each specific cell type. Two main groups of analytes are evident within the clustered heatmap. Group 1 analytes have higher levels in OCCs than OCSCs across all samples. This group consists mostly of amino acids and carbohydrates. Again, interestingly, putrescine, fumarate, gamma-aminobutyric acid, glutamate, aspartate, and proline (metabolites involved in the arginine and proline pathway discussed below) all fall within group 1 of intracellular metabolites. Group 2 metabolites have higher levels in OCSCs and lower levels in OCCs across all samples, including numerous aliphatic compounds. There were also smaller clusters of metabolites whose levels varied with time (increasing or decreasing) in one cell line but did not substantially vary in the other cell line (see S2 Fig.). Similar clustering results were obtained in analyses of extracellular metabolite levels.

### Gene set and metabolic pathway enrichment analysis results show strong concordance

Using metabolite pathway enrichment analysis (MPEA) in MetaboAnalyst, 36 pathways were found to be significantly enriched for intracellular metabolic differences between OCCs and OCSCs, listed in Table 2. Gene set enrichment analysis (GSEA) revealed 11 KEGG pathways significantly enriched in OCSC phenotype, listed in Table 3. Out of those 11 pathways, only six include metabolic reactions that convert endogenous metabolites, four of which overlapped with the pathway results from MPEA: (i) arginine and proline metabolism, (ii) fructose and mannose metabolism, (iii) primary bile acid biosynthesis, and (iv) steroid hormone biosynthesis. This close overlap in the GSEA and MPEA results suggests that changes at the transcriptional level for metabolic enzymes in OCSCs also generally manifest at the metabolite level, and supports the validity of the differences identified in each level of analysis. However, these results also suggest that in addition to the metabolic changes correlated with transcriptional levels, there are also metabolic changes that are likely mediated post-transcriptionally, since 32 pathways are enriched metabolically that are not enriched transcriptionally. Lack of concordance between transcriptional and metabolite (and potentially also protein and metabolic flux) levels is a widely-known phenomenon, and so it is not a surprise that some changes were only evident in one type of analysis.

**Table 2:** Significantly enriched KEGG pathways determined using metabolite pathway enrichment analysis.

**KEGG Pathway**	**p value**	**FDR**
Butanoate metabolism	3.19E-11	1.37E-09
beta-Alanine metabolism	1.84E-10	3.95E-09
Arginine and proline metabolism	1.32E-09	1.89E-08
Alanine, aspartate and glutamate metabolism	1.97E-09	2.12E-08
Tyrosine metabolism	1.05E-08	9.07E-08
Phenylalanine metabolism	4.50E-08	3.22E-07
Citrate cycle (TCA cycle)	5.36E-08	3.29E-07
Aminoacyl-tRNA biosynthesis	4.60E-07	2.47E-06
Nicotinate and nicotinamide metabolism	7.96E-07	3.80E-06
Glutathione metabolism	2.96E-06	1.27E-05
Lysine degradation	4.76E-06	1.71E-05
Lysine biosynthesis	4.39E-06	1.71E-05
Fructose and mannose metabolism	8.70E-06	2.88E-05
Nitrogen metabolism	3.32E-05	1.02E-04
Histidine metabolism	3.87E-05	1.11E-04
Glycerolipid metabolism	4.80E-05	1.29E-04
Primary bile acid biosynthesis	5.48E-05	1.39E-04
Valine, leucine and isoleucine biosynthesis	6.83E-05	1.47E-04
Porphyrin and chlorophyll metabolism	6.68E-05	1.47E-04
Valine, leucine and isoleucine degradation	6.83E-05	1.47E-04
D-Glutamine and D-glutamate metabolism	7.62E-05	1.56E-04
Glyoxylate and dicarboxylate metabolism	1.12E-04	2.18E-04
Biotin metabolism	1.67E-04	3.12E-04
Pantothenate and CoA biosynthesis	2.06E-04	3.69E-04
Glycerophospholipid metabolism	2.77E-04	4.76E-04
Steroid hormone biosynthesis	3.94E-04	6.52E-04
Glycine, serine and threonine metabolism	8.93E-04	1.32E-03
Pyrimidine metabolism	8.90E-04	1.32E-03
Cyanoamino acid metabolism	8.93E-04	1.32E-03
Purine metabolism	1.89E-03	2.63E-03
Thiamine metabolism	1.89E-03	2.63E-03
Pyruvate metabolism	2.93E-03	3.86E-03
Galactose metabolism	2.96E-03	3.86E-03
Cysteine and methionine metabolism	4.05E-03	5.12E-03
Methane metabolism	7.23E-03	8.89E-03
Amino sugar and nucleotide sugar metabolism	1.60E-02	1.91E-02

**Table 3:** Gene set enrichment analysis: KEGG pathways significantly enriched in OCSC phenotype.

**KEGG Pathway**	**p value**	**FDR q-val**
Fructose and mannose metabolism	0.00000	0.01334
Metabolism of xenobiotics by cytochrome P450	0.00000	0.07636
Renin-angiotensin system	0.00960	0.09388
Glycosaminoglycan biosynthesis-keratan sulfate	0.00606	0.10337
Starch and sucrose metabolism	0.01152	0.10737
ABC transporters	0.00191	0.11004
Drug metabolism-other enzymes	0.00971	0.11724
Primary bile acid biosynthesis	0.01200	0.12187
Arginine and proline metabolism	0.01304	0.12491
PPAR signaling pathway	0.00378	0.13570
Steroid hormone biosynthesis	0.01232	0.15972

**Fig 3:**
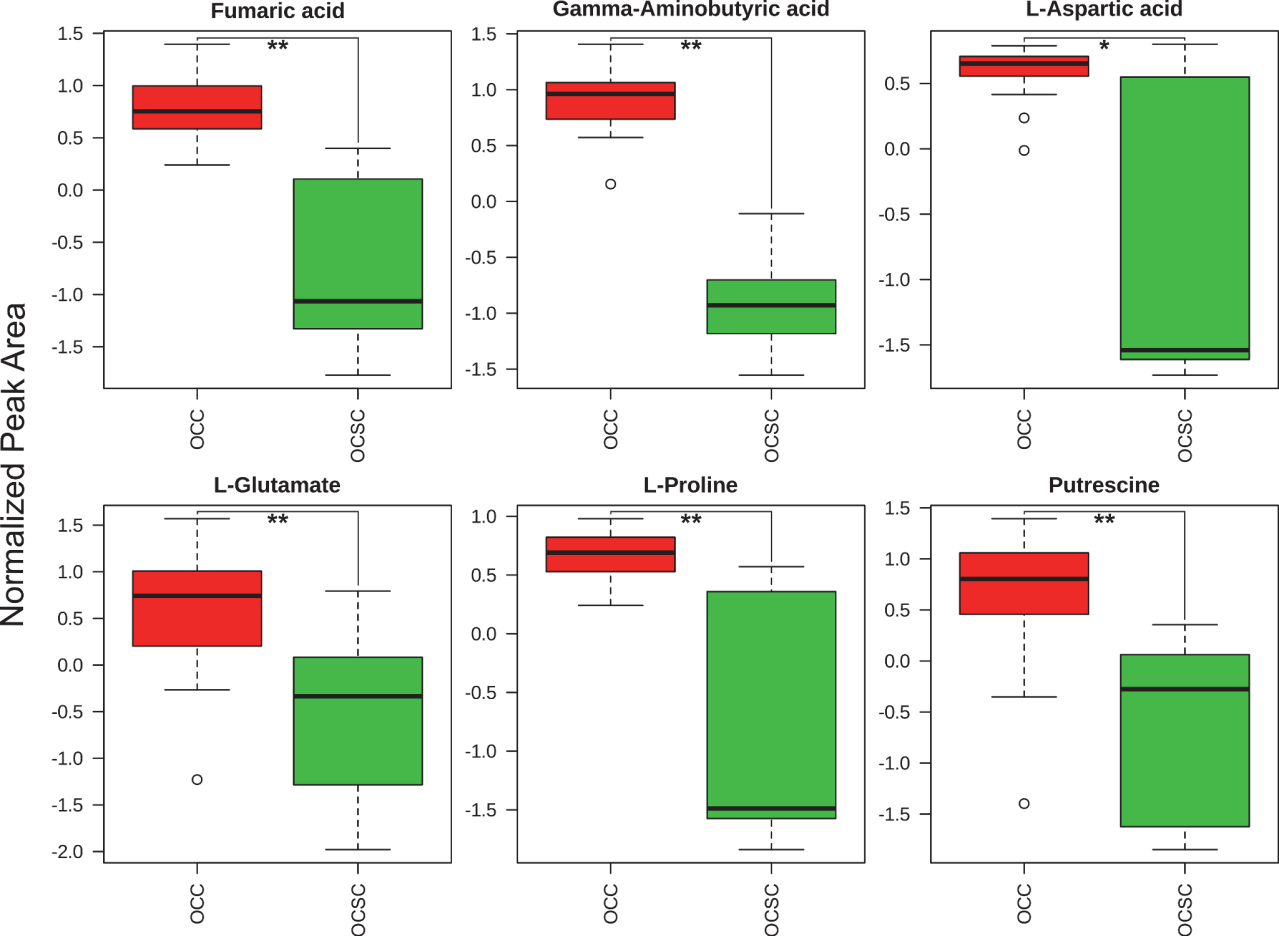
Metabolites in arginine and proline metabolism differ significantly between OCCs and OCSCs. All of the identified metabolites within the arginine and proline pathway were significantly (**: FDR < 0.0005, *: FDR < 0.005) depleted in OCSCs. Box and whisker graphs depict the normalized peak area differences between the two cell types: dark black lines are the median, boxes identify the middle 50% values, dashed lines show two standard deviation bounds, and circles indicate outliers.

One of the pathways enriched in both analyses, arginine and proline metabolism, was particularly interesting based on the number of metabolites found within the pathway that were each indvidually statistically significantly different in OCSCs as compared to OCCs. All six of the metabolites measured from this pathway (aspartate, fumarate, glutamate, gamma-aminobutyric acid, proline, and putrescine) were lower in OCSCs than OCCs, as seen in Fig. 3. Two of these metabolites, proline and putrescine, demonstrated strong concordance between their levels and the transcriptional levels of corresponding enzymes immediately upstream and downstream in the metabolic pathway [8], as shown in S3 Fig., justifying a closer look at the role that these two metabolites can play in cancer and cancer stem cells. In order to further confirm the identity of proline and putrescine, pure standards were run on the GCxGC-MS and compared to the intracellular samples. The retention times and mass spectra of the two standards were consistent with those of the annotated metabolites measured in the experimental samples.

### Proline & putrescine have been implicated in cancer

The observed changes in the levels of a number of metabolites in the arginine and proline metabolism pathway are consistent with previously-known information about the roles of these molecules in cells. For example, putrescine is a polyamine, a class of molecules that has been shown to affect numerous processes in normal and cancer cells, including proliferation, apoptosis, cell-cell interactions, and angiogenesis [39–41]. Total polyamine levels are higher in highly proliferative cells, like cancer cells, and lower in cells with low proliferation rates [40]. (However, we note that the medium control experiment, which induced significantly slower growth in OCCs, did not induce a change in putrescine levels, suggesting that a decrease in proliferation is not necessarily the cause of decreased putrescine levels.) Polyamine catabolism has been implicated in cancer, as the process can cause DNA damage and mutation via production of reactive aldehydes, production of reactive oxygen species (ROS), or depletion of free radical scavengers [42]. Many cancer therapeutics have been targeted toward enzymes in the polyamine metabolism pathway, with promising initial results [42, 43].

Likewise, proline metabolism is important to cellular processes, both in its catabolism and anabolism, and has been implicated in cancer [44–46]. During periods of high stress, proline catabolism by proline dehydrogenase/oxidase (PRODH/POX) is induced to help rescue the cell through energy production or production of ROS to induce autophagy. Proline anabolism from glutamate has been implicated in cancer via its control by the transcription factor MYC, which represses PRODH/POX expression though microRNA control and induces expression of P5CS and PYCR, enzymes that convert glutamate to proline [47]. Knockdown of P5CS or PYCR was shown to inhibit anaerobic glycolysis, thus indicating that proline anabolism from glutamate contributes to establishment of the Warburg effect [44].

### An interesting hypothesis: metabolic differences may be related to stem-like phenotypes

With both proline and putrescine and their respective pathways known to be already altered in cancer compared to normal tissues, it is perhaps surprising to find that both of these metabolites are now altered again in OCSCs relative to OCCs. Just as increased glycolytic flux is an important part of the cancerous phenotype, it is possible that the reversal of proline and putrescine accumulation in OCSCs may play a role in the stem-like phenotype. Proline has recently been shown to induce differentiation in mESCs towards an epiblast stem cell state, induced by catabolism of proline to pyrroline-5-carboxylate [48, 49]. In addition, proline has been suggested to act as a signaling molecule that controls stem cell behavior [50]. Putrescine has long been known to promote differentiation [51–54], as have other polyamines [55, 56]. Polyamines were found to be statistically significantly reduced in induced pluripotent stem cells compared to fibroblasts (although also compared to embryonic stem cells), suggesting potential involvement of polyamine metabolism in reprogramming for stem-like phenotypes [57]. This evidence together suggests the possibility that the levels of proline and putrescine, while each implicated in cancer, may play additional roles in the stem-like state of OCSCs; specifically, maintenance of one or both of these molecules at lower levels may help to maintain the stem-like state of OCSCs, consistent with ideas proposed elsewhere about the interplay between metabolism and cancer stemness [58, 59]. Additional detailed experiments are necessary to investigate this hypothesis.

### Comparison of OCSC diffferences to other cancer stem cell studies

An interesting picture emerges when differences in metabolite abundances between OCCs and OCSCs are compared with differences in metabolite abundances between ovarian borderline tumors and invasive ovarian carcinomas. While tissue concentrations of glycine, proline, glutamate, and fumarate are higher in invasive ovarian carcinomas relative to non-invasive borderline tumors [60], our data show that OCSCs display lower concentrations of these metabolites relative to OCCs. Glycine plays a role in rapid proliferation of cancer cells via *de novo* purine synthesis [10] and the SOG pathway [61]. The SOG pathway consists of serine synthesis, one-carbon metabolism, and the glycine cleavage system and supports rapid proliferation through energy production. Considering the role of glycine in rapid proliferation of cancer cells, the role of fumarate in the malignant phenotype via aberrant activation of hypoxia response pathways [62], and the role of glutamate in anabolic processes and replenishing of the tricarboxylic acid cycle intermediates during cell growth (anaplerosis) [63], it is somewhat intriguing that OCSCs are metabolically more similar to indolent and relatively benign borderline tumor cells with respect to these cancer-relevant metabolites. We hypothesize that certain phenotypic similarities between cancer stem cells and cells with less malignant potential (or non-malignant cells), might be associated with quiescence of these cell types and might play an important role in failure of cancer therapies designed to target aggressively growing clinical cancers. Similarly, our results imply that OCSCs may be less dependent on polyamines than their more differentiated progeny, which could be a more broadly applicable property of quiescent cancer stem cells that can explain the general failure of inhibitors of polyamine synthesis in clinical cancer trials [41].

Our results are also partially consistent with a recent metabolomics study of glioma-stem cells (GSCs), specifically the cultured GSCs (CGSCs). The *in vivo* tumors formed from CGSCs were noted to have increased concentrations of glycine and choline-containing compounds and decreased concentrations of glutamine, glutamate, taurine, and total creatine [16]. In our results, we did not detect choline-containing compounds, taurine, or creatine. We did, however, notice decreasing trends in both glycine and glutamate. One caveat on comparing these two experiments, though, is that the CGSCs were compared to normal tissue, while our OCSCs were compared to cancerous cells.

### Limitations

In this work we did not use control cells that would represent cells of origin or nonmalignant counterparts of epithelial ovarian cancer cells, since our primary interest was the identification of metabolic differences that can explain other biological differences between OCSCs and their more differentiated progeny (e.g. quiescence and drug resistance) rather than cancer vs. normal metabolomic differences that have already been more widely examined. Furthermore, the cell of origin of epithelial ovarian cancers is still debated, with multiple theories attempting to explain observed morphologies and phenotypes; at any rate, there is significant evidence suggesting that the cell of origin of high-grade serous epithelial ovarian cancer (the most clinically relevant and most prevalent form, for which OVCAR-3 is a model), is not ovarian surface epithelium [64]. The more widely suggested cells of origin are the endometrium and the epithelium of the (distal) fallopian tube [64, 65]. Identification of a single “normal” cell type may thus be impossible, and use of available immortalized ovarian surface epithelial cells could be misleading for interpretations. Thus, the strength of our work is in that we compare the metabolism of isogenic cancer cell lines that differ only in their cell stemness and differentiation status, no matter what the cell of origin may truly be.

We also note that the extent of biological interpretation of our data has been limited by the content of available databases and by conservative data processing decisions meant to increase confidence in the results. The metabolites used in our data analyses are not only limited by our ability to establish the biochemical identities of metabolites measured by our instrument using mass spectral databases, but also by the incompleteness of databases available for pathway-level analysis. We have also used a high similarity threshold during chromatogram processing to ensure that, when a metabolite is assigned a name, we have high confidence that the assigned name accurately reflects the metabolite’s identity. Based on these conservative decisions, we have thus potentially omitted other significant metabolites that did not meet confidence thresholds or were not available in the databases we used. Thus, there may be further support for any of the pathways discussed here, or potentially even for other pathways, as being important to OCSC metabolism; only with further refinement of pathway databases and analytical tools can a more thorough biological analysis be performed.

Finally, this work only studied one specific cancer cell line and its isogenic cancer stem cell line. It would be desirable to perform similar analyses for multiple isogenic cancer stem cell/cancer cell line pairs, as cancer is well-known to be a heterogeneous disease. However, there are very few ovarian cancer stem cell lines in existence, and none available in repositories for purchase. As such, the next steps in this work would be to derive or acquire more of such cancer stem cell lines to verify the broad applicability of the results obtained here.

## Conclusions

Overall, we have shown that an OCC line and its derived OCSCs have different metabolic profiles that are consistent with predictions based on previously observed transcriptional profiles. Both multivariate and univariate data analyses indicate that there are many significant differences in the metabolism of these two isogenic cell types; however, none of these differences were previously known. Both metabolic and transcriptional analyses of OCCs and OCSCs revealed the arginine and proline metabolism pathway as behaving differently in the two cell types, suggesting it may play a role in their phenotypic differences. The roles that two metabolites in that pathway, proline and putrescine, were previously known to play in cancer and in stem cells suggest an interesting hypothesis for their potential roles in the maintenance of the OCSC phenotype to balance proliferation and stemness. Taken together, this is the first-ever study demonstrating metabolic differences between ovarian cancer stem cells and their more differentiated isogenic progeny cells, with widespread metabolic differences suggesting at least different behaviors, if not a potential role, for metabolism in an ovarian cancer stem cell line.

## Supporting Information

S1 Fig Flow cytometry demonstrates high purity of each cell type. Red lines represent spheroid-derived cancer stem cells; green lines represent the OVCAR-3 cell line. Solid lines represent staining for CD44, a cancer stem cell marker; dotted lines represent an isotype control. The two cell types exhibit distinct CD44 expression patterns, with OVCAR-3 being CD44-negative and the OCSCs being CD44-positive. Populations are relatively pure; there is no secondary peak in either cell type to suggest substantial impurities even though the peaks for each cell type are more broad than isotype controls. The OCSCs are a fairly pure population, with at least 85% of the cells being CD44-positive.
(PNG)

S2 Fig Time series ordered hierarchial clustering shows clusters of metabolites with levels that increase or decrease with time. Columns represent time series ordered samples, color-coded according to cell type and time point. Rows represent hierarchically clustered analytes. Metabolite levels are mean-centered and unit-variance on a per-metabolite basis. Three intracellular metabolite clusters show clear temporal dependence. In group 1, OCC analyte levels are consistently high while OCSC analyte levels increase after 30 minutes. Group 2 analyte levels are consistently higher in OCCs while OCSCs start high and then start to decrease at 8 hours. Group 3 analytes are high in OCSCs while they decrease over time in OCCs.
(PNG)

S3 Fig Metabolic and transcriptional differences between OCCs and OCSCs in arginine and proline metabolism. Differences are shown on the modified KEGG arginine and proline metabolism pathway (pathway is modified to show only reactions involved in human metabolism). Boxes with red and blue outlines are genes up-regulated and down-regulated, respectively, in OCSCs as compared to OCCs. Metabolites with blue ovals have lower levels in OCSCs compared to OCCs.
(PNG)

S1 Protocol GCxGC—MS methods. Detailed description of methods used for autosampler, gas chromatograph, and mass spectrometer during sample analysis.
(DOCX)

S2 Protocol Removal of growth media effects. Methods and results for removal of growth media effects from the OCC and OCSC intracellular and extracellular data.
(DOCX)
