# Supplementary figures and images for: OVCAR-3 Spheroid-Derived Cells Display Distinct Metabolic Profiles
Source: PLoS One. 2015 Feb 17;10(2):e0118262. doi: 10.1371/journal.pone.0118262 (PMC4331360; doi:10.1371/journal.pone.0118262)

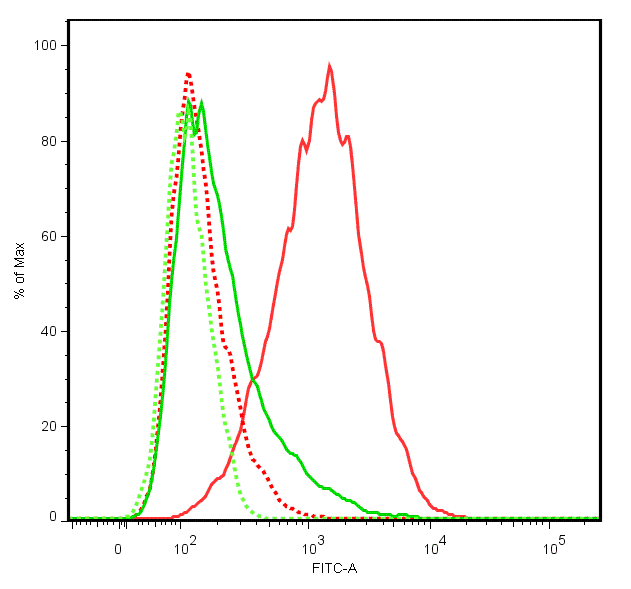

Supplement: S1 Fig — Red lines represent spheroid-derived cancer stem cells; green lines represent the OVCAR-3 cell line. Solid lines represent staining for CD44, a cancer stem cell marker; dotted lines represent an isotype control. The two cell types exhibit distinct CD44 expression patterns, with OVCAR-3 being CD44-negative and the OCSCs being CD44-positive. Populations are relatively pure; there is no secondary peak in either cell type to suggest substantial impurities even though the peaks for each cell type are more broad than isotype controls. The OCSCs are a fairly pure population, with at least 85% of the cells being CD44-positive. (PNG) [file pone.0118262.s001.png]

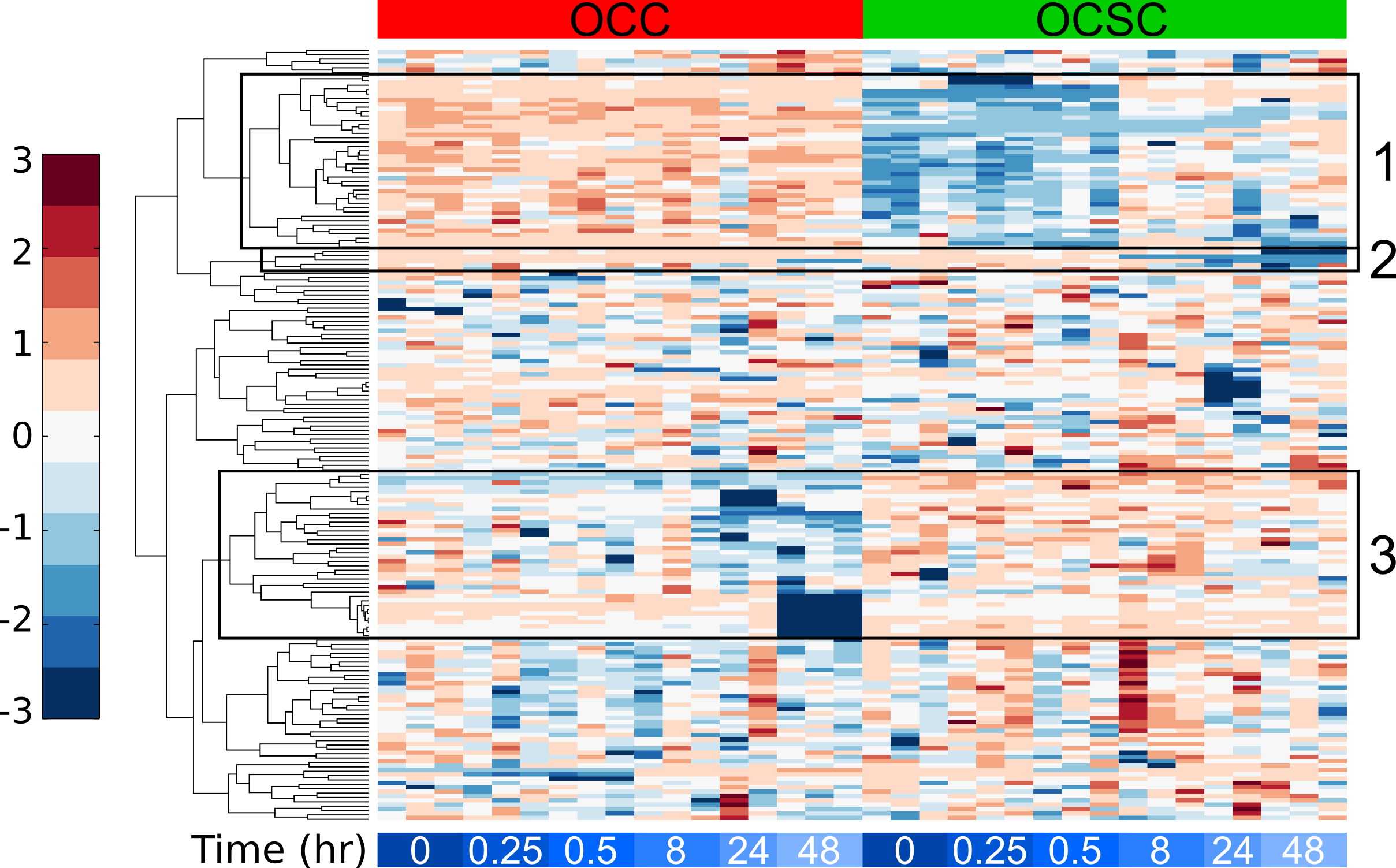

Supplement: S2 Fig — Columns represent time series ordered samples, color-coded according to cell type and time point. Rows represent hierarchically clustered analytes. Metabolite levels are mean-centered and unit-variance on a per-metabolite basis. Three intracellular metabolite clusters show clear temporal dependence. In group 1, OCC analyte levels are consistently high while OCSC analyte levels increase after 30 minutes. Group 2 analyte levels are consistently higher in OCCs while OCSCs start high and then start to decrease at 8 hours. Group 3 analytes are high in OCSCs while they decrease over time in OCCs. (PNG) [file pone.0118262.s002.png]

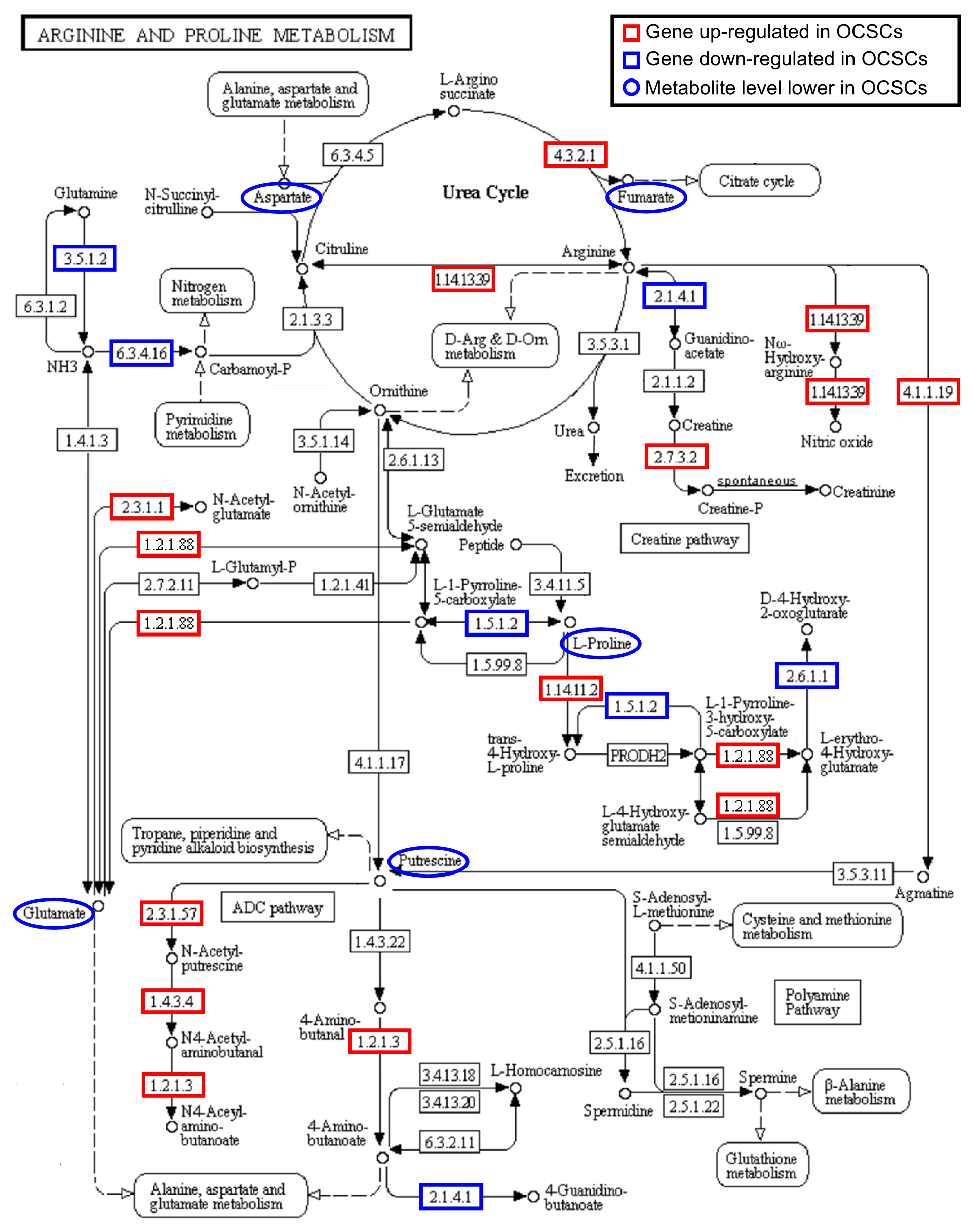

Supplement: S3 Fig — Differences are shown on the modified KEGG arginine and proline metabolism pathway (pathway is modified to show only reactions involved in human metabolism). Boxes with red and blue outlines are genes up-regulated and down-regulated, respectively, in OCSCs as compared to OCCs. Metabolites with blue ovals have lower levels in OCSCs compared to OCCs. (PNG) [file pone.0118262.s003.png]
