# Supplementary material for: OVCAR-3 Spheroid-Derived Cells Display Distinct Metabolic Profiles
Source: PLoS One. 2015 Feb 17;10(2):e0118262. doi: 10.1371/journal.pone.0118262 (PMC4331360; doi:10.1371/journal.pone.0118262)
Supplement: S1 Protocol — Detailed description of methods used for autosampler, gas chromatograph, and mass spectrometer during sample analysis. (DOCX) [file pone.0118262.s004.docx]

# Additional File 1: GCxGC-MS Methods

## AS Method

An Agilent 7683 autosampler was used. Three pre-washes with pyridine were performed before each injection. The sample was then pumped into the syringe 4 times. The syringe size was 10 µL with a sample volume of 1 µL injected into the inlet. Three post-washes with pyridine were performed after injection.

Table 1: Main Oven Temperature Programs

| Rate (°C/min) | Target Temp (°C) | Duration (min) |
| --- | --- | --- |
| Intracellular Samples | | |
| Initial | 50 | 1 |
| 10 | 215 | 0 |
| 5 | 240 | 0 |
| 10 | 310 | 2 |
| Extracellular Samples | | |
| Initial | 50 | 1 |
| 10 | 220 | 0 |
| 1 | 225 | 0 |
| 10 | 310 | 2 |

## GC Method

An Agilent 7890 gas chromatograph adapted to GCxGC analysis was used. The first column was an HP-5 30m x 0.320 mmID x 0.25 µm and the second was Rtx-200 2m x 0.180 mmID x 0.20 µm. The excluded masses in auto mass defect mode option was chosen. Helium was used as the carrier gas with a corrected constant flowrate of 1.00 mL/min. An inlet septum purge flow of 3mL/min was chosen. The inlet was operated in splitless mode with a purge flow of 100 mL/min set to start 30 seconds after injection, yielding a total flow of 101 mL/min. Gas saver mode was used, with a flow of 20 mL/min set to start a minute after injection.

Table 2: Modulation Timing

| # | Start (s) | End (s) | Modulation Period (s) | Hot Pulse Time (s) | Cold Pulse Time (s) |
| --- | --- | --- | --- | --- | --- |
| Intracellular Samples | | | | | |
| 1 | Start | 648 | 2.00 | 0.25 | 0.75 |
| 2 | 648 | 698 | 2.50 | 0.40 | 0.85 |
| 3 | 698 | 896 | 2.25 | 0.60 | 0.53 |
| 4 | 896 | End | 2.00 | 0.60 | 0.40 |
| Extracellular Samples | | | | | |
| 1 | Start | 650 | 2.00 | 0.30 | 0.70 |
| 2 | 650 | 700 | 2.50 | 0.30 | 0.95 |
| 3 | 700 | 909.25 | 2.25 | 0.40 | 0.72 |
| 4 | 909.25 | 1099.25 | 2.00 | 0.50 | 0.50 |
| 5 | 1099.25 | 1349.25 | 2.00 | 0.80 | 0.20 |
| 6 | 1349.25 | 1445.25 | 3.00 | 1.10 | 0.40 |
| 7 | 1445.25 | End | 2.00 | 0.80 | 0.20 |

The main oven temperature program can be found in Table 1. The secondary oven temperature offset was 10°C and the modulator temperature offset was 35°C from the main oven. An equilibration time of 60 seconds was set for the ovens. The modulation timing is listed in Table 2. The transfer line was set to 320°C for the entire run.

## MS Method

A LECO Pegasus IVD time of flight mass spectrum (TOF-MS) was used. The total MS method time was based on the GC method time. The acquisition delay was set to 400 seconds, with the filaments being turned off until then. The collection mass range was from 50 to 500 u. The acquisition rate was set to 200 spectra/second. The detector voltage was set to 100 V above the optimized voltage with the electron energy set to -70 V. The mass defect mode was set to manual with the mass defect 0 mu/ 100 u. The ion source temperature was 220°C and the run had to wait for the ion source temperatures to reach the set point before starting acquisition.
