# Supplementary material for: OVCAR-3 Spheroid-Derived Cells Display Distinct Metabolic Profiles
Source: PLoS One. 2015 Feb 17;10(2):e0118262. doi: 10.1371/journal.pone.0118262 (PMC4331360; doi:10.1371/journal.pone.0118262)
Supplement: S2 Protocol — Methods and results for removal of growth media effects from the OCC and OCSC intracellular and extracellular data. (DOCX) [file pone.0118262.s005.docx]

# Additional File 2: Removal of growth media effects

## Media control experiment

To control for the differences in media, a secondary experiment was performed where ovarian cancer cells (OCCs) were grown in parallel in their normal media and ovarian cancer stem cell (OCSC) media for 48 hours. Intracellular and extracellular samples were taken at 0, 24, and 48 hours in the same manner as described above for the OCCs. Cell counts were also taken (data not shown) and it was discovered that OCCs grow slower in the OCSC medium than they do in their normal medium, mimicking the slower growth rate of OCSCs. Intracellular and extracellular samples were analyzed on the GCxGC-MS with the OCC and OCSC samples, and all data were processed together.

## Removal of growth media effects from data sets

To specifically identify media effects, MetaboAnalyst was first used to analyze only the media control samples, as described in the main text. Any metabolites found to have significant differences (t-test, all time points, FDR < 0.05) between the OCCs grown in OCC media and the OCCs grown in OCSC media were then removed from the main data set to eliminate metabolic changes due to media differences. This was done for both the intracellular and extracellular data sets.

## Metabolic differences due to growth in different media were succesfully removed from intracellular samples

OCSCs are grown in different media than OCCs. Since growth in different media could cause metabolic changes and confound the metabolic differences seen between the two cell types, a media control experiment was performed to allow for removal of metabolic changes due purely to media effects. Principal components analysis (PCA) showed that before removal of the media effects, the samples from the two different media types separated in principal component (PC) 2, seen in Figure 1A. Even though separation is seen, it is still less than the overall separation between OCCs and OCSCs, meaning it represents less of the overall variance in the dataset. Once the media effects were removed, no PCA-based separation is evident between OCCs grown in the two different media while the differences between OCCs and OCSCs are still just as prominent (Figure 1B), showing that the differences between OCC and OCSC intracellular metabolism are not simply due to growth in different media. These intracellular analytes are the basis for all of our biologically-related discussion, and so any differences in these analytes between OCCs and OCSCs should not be attributed to medium differences.

In the extracellular dataset, a large difference between the OCCs in OCC media and OCCs in OCSC media samples was seen before removal of the statistically different analytes, as seen in Figure 2A. After removal, there is still separation visible between the two media control samples (Figure 2C) in the metabolite-only data. This separation is less than that seen between the OCC and OCSC extracellular samples. Though we believe these differences still support the results of our intracellular analyses, since the media effect was not completely removed from the extracellular samples, we focused only on the intracellular samples for resultant analyses and discussion.


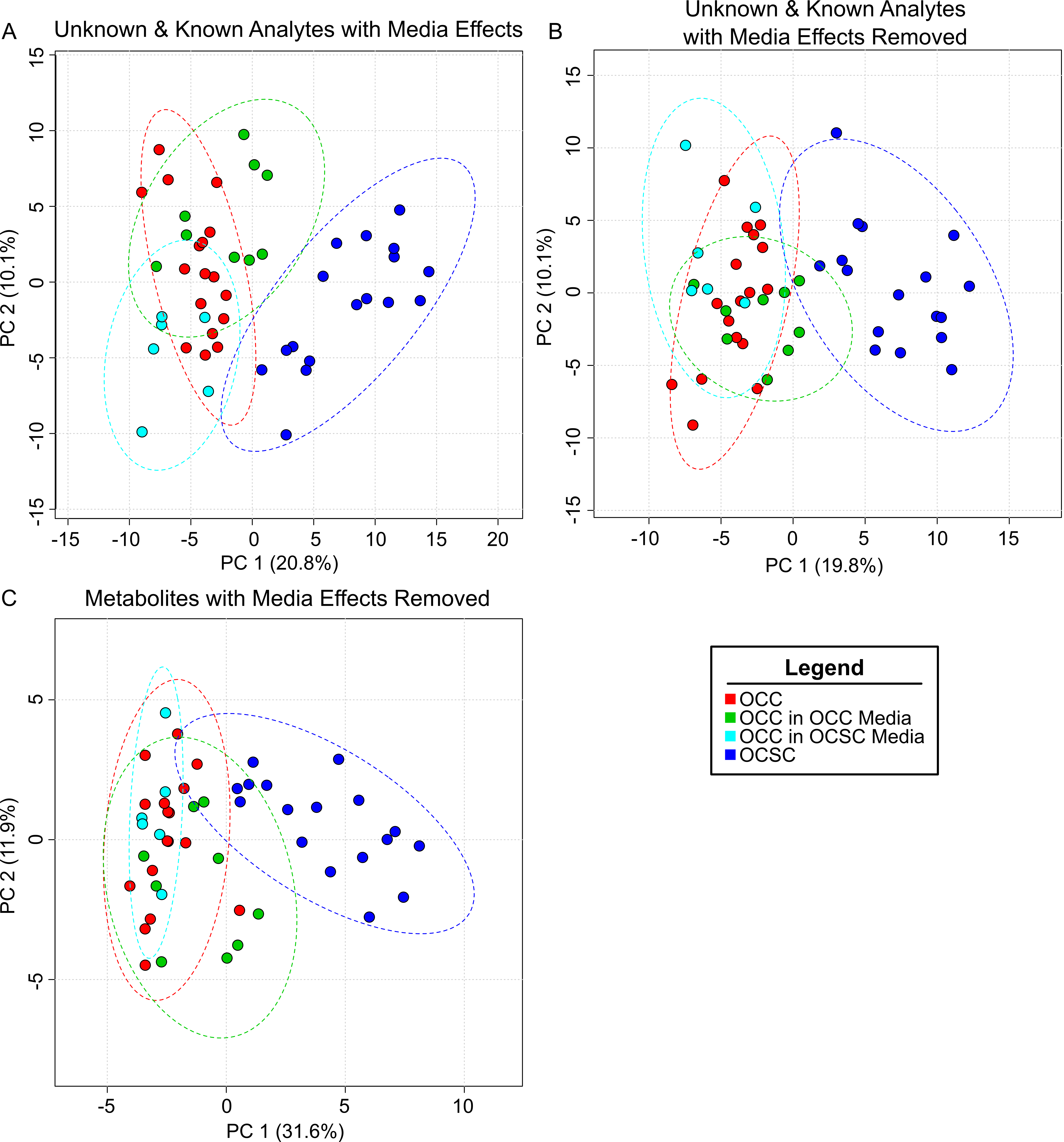


Figure 1: Intracellular media effects are successfully removed from OCC vs OCSC comparisons. Principal components analysis of OCC (red), OCSC (blue), and the two media control (OCCs grown in OCC media (green) and OCSC media (cyan)) intracellular samples. (A) Media effects are clearly seen in PC 2 through the separation of two media control samples. (B) and (C) After removing media effects, the two media control samples are overlapping, showing that there are no visible media effects in the unknown and known (B) or metabolite-only (C) intracellular analyses.


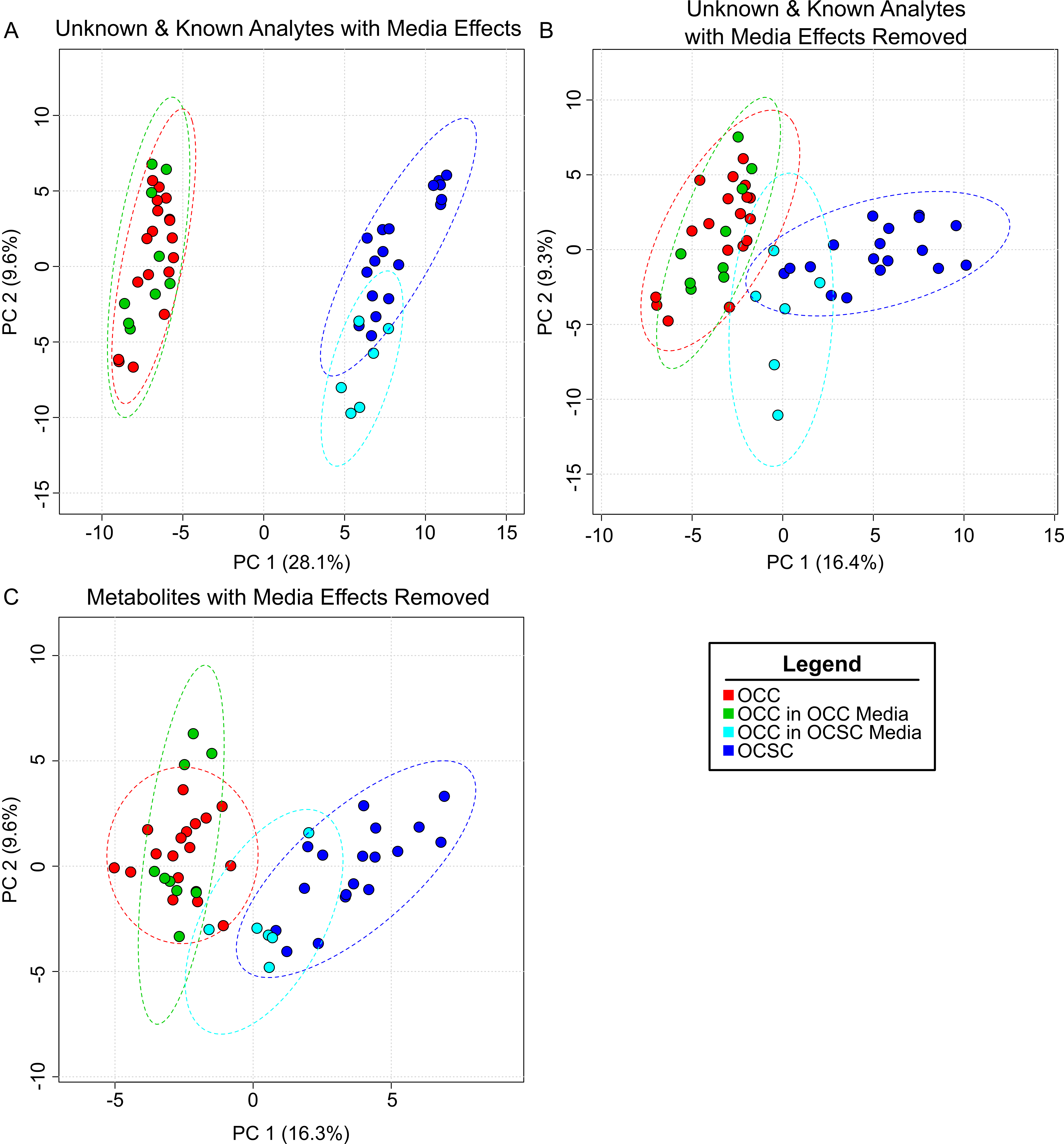


Figure 2: Extracellular media effects cannot be removed through simple removal of statistically different analytes. Principal components analysis of OCC (red), OCSC (blue), and the two media control (OCCs grown in OCC media (green) and OCSC media (cyan)) extracellular samples. (A) Media effects are clearly seen in PC 1 through the separation of two media control samples. (B) and (C) After removing media effects, the two media control samples are closer, showing the reduction of media effects but not complete removal in the unknown and known (B) and metabolite-only (C) extracellular analyses.
